# Management of LH/FSH deficiency among assisted reproduction specialists in Spain: a Delphi consensus

**DOI:** 10.3389/fendo.2025.1498062

**Published:** 2025-08-05

**Authors:** Sonia Lobo, Beatriz Álvaro, Joana Peñarrubia, Carlos Ignacio García Fernández, Elisa Gil, Joaquín Llácer

**Affiliations:** ^1^ Human Reproduction Unit, Hospital La Paz, Madrid, Spain; ^2^ Reproductive Medicine Service, Hospital de Sant Pau y Fundació Puigvert., Barcelona, Spain; ^3^ IVI-RMA, Reproductive Medicine, Barcelona, Spain; ^4^ Merck S.L.U., Medical Affairs, An Affiliate of Merck KGaA Darmstadt Germany, Madrid, Spain; ^5^ IVI-RMA, Reproductive Medicine, Zaragoza, Spain; ^6^ Ginefiv-GeneraLife, Reproductive Medicine, Madrid, Spain

**Keywords:** luteinizing hormone, follicle-stimulating hormone, deficiency, Delphi, consensus

## Abstract

**Background:**

Luteinizing hormone (LH) and follicle-stimulating hormone (FSH) deficiency can decrease women fertility, due to compromised gametogenesis and steroidogenesis. Several factors, like age, LH/FSH and their receptor polymorphisms, and gonadotrophin-releasing hormone analogue protocols; might result in a hypo-response to ovarian stimulation. The International Committee for Monitoring Assisted Reproduction Technologies (ICMART) highlighted the importance of addressing LH/FSH deficiency for a successful outcome. The aim was to understand if consensus exists on LH/FSH deficiency management among specialists in assisted reproduction based on the latest available evidence.

**Methods:**

An online, two-round Delphi consensus questionnaire was conducted from December 2021 to January 2022. The questionnaire comprised 21 statements concerning the action of LH/FSH in disrupted physiological conditions, clinical presentation of LH/FSH deficiency and its importance in assisted reproduction. A 70% agreement threshold was established for each statement.

**Results:**

Responses from 45 and 42 assisted reproduction Spanish specialists were gathered in the first and second rounds. Consensus was reached in almost half (10/21) of the statements. Participants mostly agreed on the relevance of LH/FSH deficiency due to a reduction of the action and production of gonadotropins, the importance of the effect of different glycosylation variants and age in LH/FSH action and of estradiol (E_2_) levels during ovulation discharge, the use of POSEIDON criteria to individualize the treatment of patient with poor prognosis, and the use of recombinant LH supplementation in low response patients.

**Conclusions:**

It is important to consider the diverse factors that can lead to LH/FSH deficiency in order to optimize its management and improve reproductive outcomes.

## 1 Introduction

Although many intra-ovarian factors are involved during the folliculogenesis process, the luteinizing hormone (LH) and follicle-stimulating hormone (FSH) play a major role during both follicle development and ovulation (1, 2). Both hormones are synthesized and secreted by the gonadotroph cells, which are located in the lateral space of the pituitary gland, in response to the pulsatile release of the gonadotrophin-releasing hormone (GnRH) (3). FSH stimulates the development of ovarian follicles through its interaction with FSH-sensitive granulosa cells of early antral follicles (4). On the other hand, the LH pulse is related to the production of steroid hormones in ovarian theca cells, since this hormone is transferred to the granulosa cells of developing follicles to trigger the production of estradiol (E_2_) (5).

Due to the key role played by these hormones in pre-antral follicle development, a deficiency either in their production or action might lead to a compromised gametogenesis and gonadal steroid production, triggering a reduction of female fertility (2, 6–8). Loss in LH/FSH production is usually due to mutations in genes that encode proteins involved in the regulation of GnRH neuronal development, migration from the nasal placode to the hypothalamus, GnRH secretion, or GnRH action (9). Conversely, LH/FSH action is subject to factors such as the frequency and amplitude of GnRH peaks, different isoforms of LH and FSH, polymorphisms of FSH and LH and their receptors, and intracellular signaling, as well as different demographic, clinical and treatment factors such as ageing, comorbidities, oral contraceptives and GnRH analogue protocols (10–14). Because of the negative reproductive outcomes linked to this condition, the International Committee for Monitoring Assisted Reproductive Technologies (ICMART) has recently emphasized the importance of gonadotropin action in determining a deficiency of both LH and FSH (15).

Success rates in assisted reproductive technologies are closely linked to both medical management (including the related technologies used during infertility treatment) and patient characteristics. Accordingly, personalized management strategies have been recently proposed to optimize the efficacy and safety outcomes in women undergoing assisted reproduction (16, 17). Guidelines such as ESHRE’s Ovarian Stimulation guideline, provide clinicians advice and best practices on ovarian stimulation, based on the evidence (18). However, deficiency of LH and FSH is not addressed in these protocols, leaving an important gap regarding the management of these patients with reduced ovarian response. Moreover, no consensus has been reached to date regarding neither the LH cut-off to identify patient subgroups requiring LH supplementation nor the definition about an optimal LH range. However, studies suggest an improved ovarian response in patients with suboptimal responses to recombinant FSH (r-FSH) therapy, advanced maternal age and at risk of poor response due to GnRH antagonists after a LH supplementation was administered (19).

Therefore, the objective of this project was to understand if consensus exists on the management of LH/FSH deficiency among the specialists in assisted reproduction who practice medicine in Spain based on the latest available evidence.

## 2 Materials and methods

### 2.1 Study design

This was a nationwide exploratory study conducted by a panel of experts which followed the Delphi methodology and consisted of two rounds of participation, conducted from December 2021 to January 2022. A scientific board, composed of five experts, was involved in the design and development of the study. These experts were members of the Reproductive endocrinology group of interest of the Spanish Society of Fertility.

The participants in the Delphi panel were selected by the scientific committee based on explicit and predefined criteria, including clinical experience in reproductive endocrinology and in the diagnosis and management of FSH/LH deficiency, number of scientific publications in the field, and citation metrics based on an external database. This ensured the inclusion of both junior and senior professionals actively involved in assisted reproduction. All selected participants were practicing gynecologists with proven expertise in reproductive medicine, and their profiles were reviewed and confirmed by the scientific committee. To ensure a diverse and representative panel, the recruitment strategy also aimed for geographical and institutional diversity. The final panel was validated by the scientific board to ensure representativeness and mitigate the risk of bias associated with homogeneous institutional protocols.

**Figure 1:**
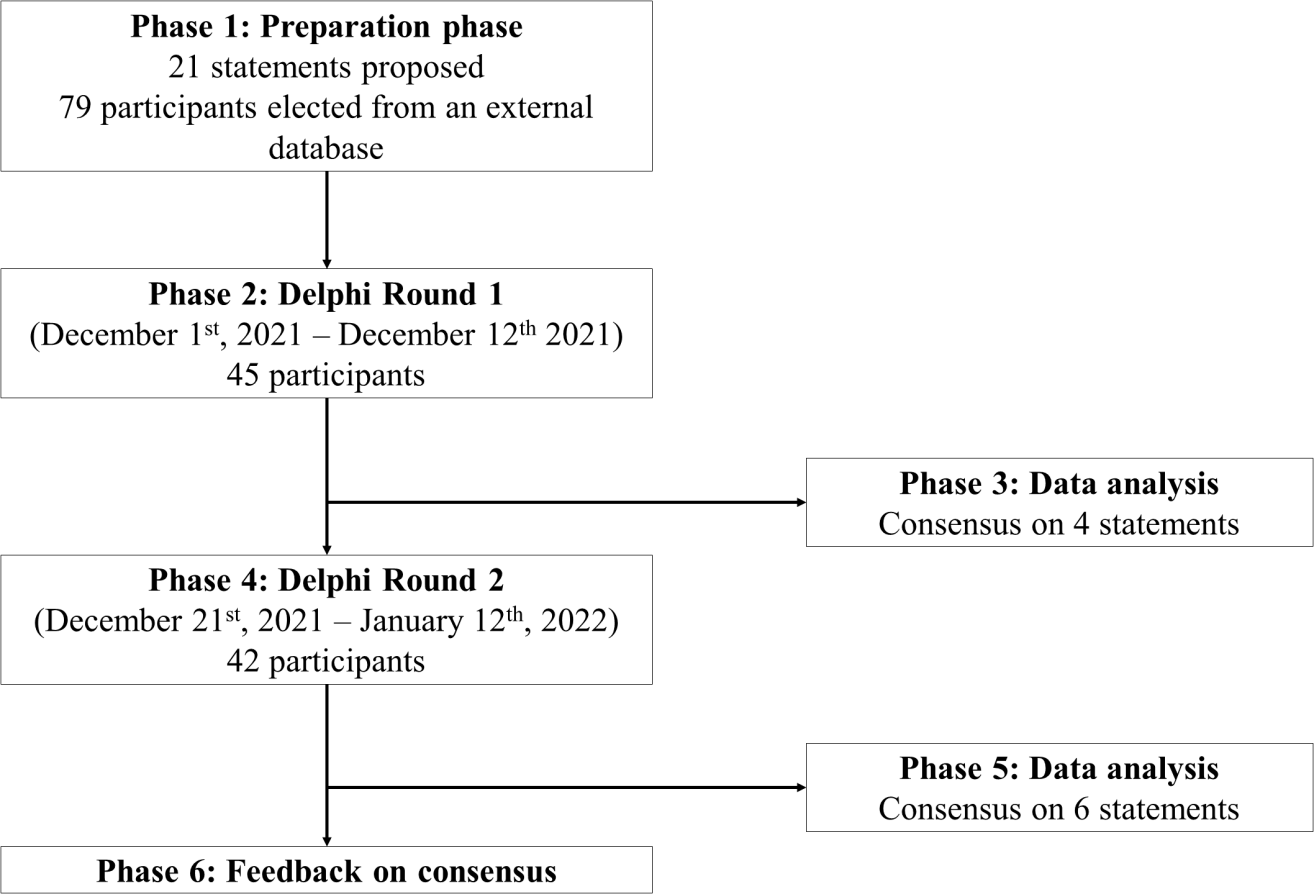
Design of the Delphi consensus process, in which the different phases, their schedule and the number of participants taking part in each of them are represented.

The project consisted of three phases. During the first one, the preparation phase, the scientific board agreed about the design of the Delphi study and the statements to be included in the questionnaire based on the available related evidence (14, 20). The second phase comprised a two round survey, which was conducted online from December 1^st^ to December 12^th^ and from December 21^st^ to January 12^th^. A scientific committee meeting was held between these rounds to discuss the obtained results. After the interpretation of the first-round results, the scientific committee decided to rephrase and implement minor wording adjustments in some of the statements in order to improve their understanding. Finally, the analysis of the results from the second round and the feedback of the consensus took place in the third phase (**Figure 1**).

### 2.2 Questionnaire

The questionnaire (**Table 1**) comprised 21 statements, grouped into two sections and seven domains. The two main sections included statements regarding the action of LH & FSH in physiologic and altered conditions and LH & FSH deficiency in assisted reproduction. Each item had a bibliographic reference attached to provide participants with additional information. A total of 79 interviewees were selected by the scientific committee according to their experience in the field of assisted reproduction, and number of scientific publications and citations based on an external database. The survey, anonymous and not remunerated, was sent by email to the persons included in the database in both rounds. Since the process was completely anonymous, participants were advised to answer the second round only if they had participated in the first one. As the survey was sent through an email campaign, in order to receive a meaningful number of responses, the survey was sent to a higher number of participants than those who were expected to complete the survey. Out of 79, a total of 45 participants answered in the first round and 42 participants answered in the second round, representing more than the 50% of the total sample of participants.

**Table 1:** Study questionnaire.

Section 1: LH and FSH action in physiology and altered conditions
Domain 1: Synergic action of LH and FSH action in the follicle
Statement 1: In my routine clinical practice, I acknowledge and consider the importance of FSH/LH deficiency due to a reduction of the action and production of gonadotropin.
Statement 2: After the administration gonadotropin-releasing hormone (GnRH) agonists/antagonists, a transient functional state of hypogonadotropic hypogonadism with a severe LH deficiency is created.
Statement 3: In my routine clinical practice, I consider the baseline levels of LH before starting the stimulation.
Statement 4: LH levels should be monitored throughout the stimulation cycle.
Domain 2: Effect of different LH and FSH glycosylation variants of LH/FSH and age in the action of LH/FSH
Statement 5: In my usual clinical practice, when selecting the gonadotropin for the stimulation protocol, I take into account the different glycosylation patterns of FSH.
Domain 3: Effect of genetic variants of LH/FSH and their receptors in the action of LH/FSH
Statement 6: In my usual practice, I consider that the polymorphisms of the FSH receptor and/or the beta variant of LH, could have a lower performance in the ovarian response.
Statement 7: In my clinical practice, I consider that the polymorphisms in the FSH and/or the beta variant of LH, could increase the risk of low response in patients with associated risk factors (advanced age, diabetes, obesity, chronic illness…).

Section 2: LH and FSH deficiency in medically assisted reproduction
Domain 4: Clinical presentation of LH and FSH deficiency
Statement 8: In my clinical practice, I consider estradiol (E2) levels at the end of the stimulation with the aim of deciding the type of triggering.
Statement 9: The presence of low E2 leves regarding follicular response are indicative of deficiency of activity produced by LH during stimulation with recombinant FSH monotherapy.
Statement 10: In my clinical practice, I consider that the use of long term contraceptives can increase the risk of hypo-response in patients with associated risk factors (advanced age, diabetes, obesity, chronic illness…).
Domain 5: Deficiency induced by the use of analogues of Gonadotropin releasing hormone (GnRH)
Statement 11: LH deficiency caused by agonists/antagonists of GnRH potentially affects folliculogenesis, steroidogenesis and oocyte competence in detriments of clinical results
Statement 12: In my clinical practice, I consider that the use of analogues of GnRH can increase the risk of hypo-response in patients with associated risk factors (advanced age, diabetes, obesity, chronic illness…).
Domain 6: Reduction in LH/FSH action in advanced maternal age in patients going through assisted reproduction techniques
Statement 13: In my clinical practice, I consider that advanced maternal age women have exacerbated gonadotropin deficiency.
Statement 14: The combined treatment of r-hFSH:r-hLH improved the results in implantation, clinical pregnancy and live birth rates in women of advanced maternal age.
Domain 7: Hyporesponse to stimulation due to reduced action of LH/FSH
Statement 15: In my clinical practice, in order to catalogue a patient as low responder to ovarian stimulation I use the FORT index (relation between the number of follicles that reach a size between 16 and 22 mm the day of HCG administration after ovarian stimulation and the number of antral follicles measured between the second and fourth day of menstruation).
Statement 16: In my clinical practice, in order to catalogue a patient as low responder I use the FOI index (index follicle-oocyte, relation between the number of retrieved oocytes and the number of antral follicles at the beginning of the ovarian stimulation).
Statement 17: In my clinical practice, I use the POSEIDON classification to individualize the treatment of patients with low prognosis.
Statement 18: The different gonadotropin schemes can affect endometrial receptivity.
Statement 19: In patients with hypogonadotropic hypogonadism, the use of r-hFSH:rhLH is superior in outcomes to human menopausal gonadotropin (hMG).
Statement 20: In situations of deficiency of production and/or action of gonadotropins (previous contraceptives, advanced maternal age…) the use of r-hFSH:rhLH is superior in outcomes to those of hMG.
Statement 21: In my daily practice I use r-LH supplementation in patients with unexpected low response after stimulation with r-FSH.

### 2.3 Determination of the degree of consensus

A five-point Likert scale was used to determine the degree of consensus for each of the statements: strongly disagree (1), disagree (2), neither agree nor disagree (3), agree (4), and strongly agree (5). A consensus of agreement was established when more than 70% of the participants either “agree” or “strongly agree” for the corresponding item, to ensure a high level of consistency among expert opinions. Conversely, a consensus of disagreement was defined when more than 70% of the interviewees either “disagree” or “strongly disagree” for the corresponding item. If neither of the two possible consensus options were met, no consensus was established for that item. Statements in which no consensus was reached after the first round were transferred to the second one, with the results obtained in the previous round being added to each item. As stated above, some of the statements from the first round were rephrased in the second round to ensure clarification and a better understanding in the interpretation of the participants.

## 3 Results

**Figure 2:**
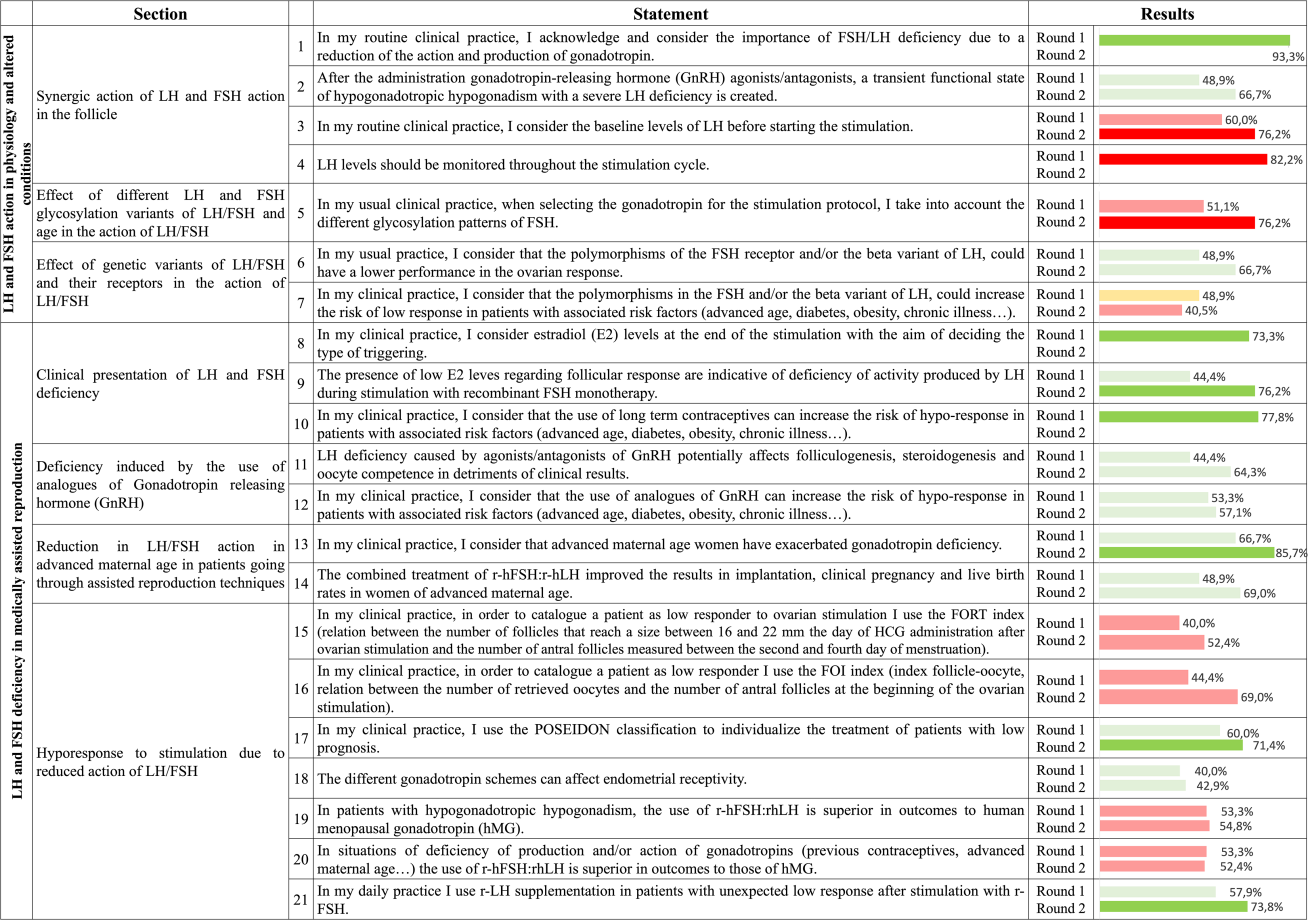
Degree of consensus for the metrics assessed in the two rounds of the Delphi method. Green represents a consensus on agreement, red represents a consensus on disagreement, and yellow represents a consensus on neither agreement nor disagreement. Light colors represent that the statement did not surpass the 70% threshold, indicating that no consensus was reached either in the overall agreement or disagreement. Conversely, strong colors represent that a consensus on the agreement or disagreement was reached. Follicle-to-Oocyte Index, FOI; follicle-stimulating hormone, FSH; Follicular Output Rate, FORT; human chorionic gonadotropin, hCG; luteinizing hormone, LH.

Responses from 45 and 42 assisted reproduction specialists from Spain were gathered in the first and second rounds, respectively. The results are summarized in **Figure 2**.

Within the first topic, the subtopic regarding the synergic action of LH and FSH in follicles was one of the subdomains in which a higher consensus was reached, since in three out of its four statements surpassed the 70% threshold. In two statements an agreement was reached and were related to the acknowledgement of LH/FSH deficiency due to a reduction of the action and production of gonadotropin (93.3% of agreement in the first round) and consideration of the baseline LH levels before starting the stimulation process. In the second statement a 76.2% of disagreement was achieved in the second round. Regarding the third item, participants disagreed about the monitorization of LH levels throughout the monitorization cycle (82.2% of disagreement in the first round). The remaining item of this subdomain, regarding the transient functional state of hypogonadotropic hypogonadism with a severe LH created after GnRH agonists/antagonists administration, was close to reach a consensus on agreement (48.9% and 66.7% of agreement in the first and second rounds). Regarding the effect of different LH and FSH glycosylation variants and female age on LH and FSH action, there was an overall disagreement in considering the different glycosylation patterns of FSH when choosing the gonadotropin for the stimulation protocol (76.2% of disagreement in the second round). Finally, no consensus was reached in neither of the two statements regarding the effect of genetic variants of LH/FSH and their receptors on the action of these two hormones, with participants almost agreeing on the statement that carriers of polymorphism in the FSH receptor and/or beta variant of LH have a lower performance of the ovarian response (48.9% and 66.7% of agreement, respectively).

Regarding the second topic, related to LH and FSH deficiency in medically assisted reproduction, a high consensus was reached in the three items assessed, with two of them exceeding the 70% threshold in the first round (73.3% for taking E_2_ levels into account when the ovulation discharge method is selected and 77.8% for considering that long-term contraceptive methods might increase the risk of hypo-responsiveness in patients with associated risk factors). In the sub-topic including statements about the LH/FSH deficiency induced by GnRH analogue protocols, neither of the two items reached a consensus. However, participants almost agreed on this condition potentially affecting folliculogenesis, steroidogenesis and ovarian competence, at the expense of the clinical results (64.3% agreement on the second round). Respecting the reduced LH/FSH action in advanced maternal age women most interviewees agreed that these patients have their gonadotropin deficiency exacerbated (66.7% and 85.7% in the first and second rounds, respectively). In the other statement, regarding the improved implantation, clinical pregnancy and live birth rates in these women through the treatment with r-hFSH:r-hLH, no consensus was reached (69.0% of agreement in the second round). Finally, only two items out of seven included in the subtopic regarding the hypo-response to ovarian stimulation due to reduced LH/FSH action, reached an agreement. These statements were referred to either the use of the patient-oriented strategies encompassing individualized oocyte number (POSEIDON) classification to individualize the treatment of patients with bad prognosis (71.4% of agreement in the second round) or the use recombinant LH (r-LH) supplementation in patients with unexpected low responses after r-FSH stimulation (73.8% of agreement in the second round). In the remaining statements, most of them leaned towards disagreement, such as the use of both the Follicular Output Rate (FORT) and Follicle-to-Oocyte Index (FOI) to classify a patient as low respondent (52.4% and 69.0% of disagreement in the second round, respectively) and the belief that the use of r-hFSH:r-hLH is superior to human menopausal gonadotropins (hMG) in patients with hypogonadotropic hypogonadism.

Overall, after the two rounds consensus was reached in almost half (10/21 items) of the statements.

## 4 Discussion

A reduced production or action of gonadotropins might result in clinically significant LH/FSH deficiency, impairing gametogenesis and steroidogenesis. LH stimulates androgen production in theca cells, while FSH supports follicular development and converts androgens to E_2_ in granulosa cells. Disruptions in GnRH pulsatility or receptor polymorphisms can disturb this hormonal balance, potentially causing a hypo-response (14). This could explain why some women show a suboptimal response to r-hFSH during ovarian stimulation despite normal gonadotropin levels and ovarian reserve in the context of medically assisted reproduction. However, even if the ICMART defined hypogonadotropic hypogonadism as a gonadal failure associated with reduced gametogenesis and reduced gonadal steroid production due to reduced gonadotropin production or action (15), current guidelines do not provide protocols to help tackle this scenario, with assisted reproduction specialists relying on their own experience. This Delphi consensus provide insights into the management practices Spanish assisted reproduction specialists follow to manage these patients.

### 4.1 Statements with consensus achievement

Consensus was achieved for 10 out of the 21 statements included in the Delphi process. These statements reflect a clear alignment in clinical practice among Spanish assisted reproduction specialists regarding the recognition and management of LH/FSH deficiency.

One of the strongest consensuses (93.3% agreement in the first round) was reached on domain 1, recognizing LH/FSH deficiency as a key factor in female infertility. This statement aligns with its role as a key component in female infertility, triggered by impaired gametogenesis and steroid production (2, 6–8). Due to its importance, even the ICMART has addressed this LH/FSH deficiency (15). According to the study by Bosch et al., reduced LH and FSH action may stem not only from congenital causes or receptor polymorphisms, but also to acquired causes (eating disorders, excessive exercise, age-related GnRH pulse deterioration), organic causes (tumors, radiotherapy, trauma or glandular surgery) or use of GnRH analogues (14). These deficiencies are observed in infertile patients with amenorrhea, who do not ovulate and often have low E_2_ or gonadotropin levels.

Regarding LH level measurement, participants reached consensus on the disagreement for its utility either before or during the stimulation (76.2% y 82.2% respectively). Ramachandran et al. reported that serum LH levels during r-FSH and GnRH antagonist IVF are not predictive of oocyte retrieval, oocyte maturation, fertilization, grade 1 embryo, implantation and clinical pregnancy rates in normogonadotropic women, suggesting LH measurement is not mandatory during IVF with this protocol (21). Similarly, Verschuere et al., observed that LH levels dropped below 1.2 IU/L in nearly half of antagonist cycles; however, this biochemical over suppression did not negatively impact pregnancy outcomes, indicating that low LH levels during stimulation may not be intrinsically detrimental and may not justify routine monitoring (22). In contrast, other studies stated that LH fluctuations during the follicular phase might be unfavorable to endometrial receptivity and pregnancy rates, with positive outcomes increasing the lower LH levels were both at baseline and hCG initiation (23). Supporting this, Luo et al. showed that low LH levels during GnRH antagonist protocols were linked to reduced cumulative live birth rate per retrieval cycle in normogonadotropic women, likely due to a detrimental effect of these low levels on fresh embryo transfer outcomes (24). Despite some studies suggesting a potential impact of low LH levels on reproductive outcomes, the overall body of evidence, reinforced by the expert consensus reached in this study, indicates that routine LH monitoring during ovarian stimulation lacks sufficient clinical justification. Routine assessment of LH levels in this setting should therefore not be considered standard practice.

In Domain 2, which addressed the impact of different glycosylation patterns on LH/FSH, a consensus of disagreement was reached (76.2% on second round) on whether these biochemical characteristics should influence gonadotropins selection. This contrasts with existing evidence suggesting that glycosylation affects protein folding, secretion, and metabolism of FSH and other gonadotropins, as well as regulation of its half-life, activity at the target cell level and signal transduction (25). Commercial gonadotropins differ in glycosylation profiles (26), and the amount of sialic acid influences receptor binding, biological activity and clearance (27). Additionally, the less acidic and short-lived h-FSH variants might produce more physiological effects when specific response endpoints are assessed (25). Notably, recent findings show that hypoglycosylated FSH variants, such as FSH21, enhance folliculogenesis and oocyte quality by promoting early cell-to-cell communication within the follicle (28). Variations in clinical outcomes such as live birth or pregnancy rates might relate to glycosylation diversity (29–32). Moreover, Conforti et al. noted that the response to gonadotropins could be influenced by some receptor and ligand polymorphisms, modifying gonadotropin-receptor affinity, influencing FSH levels and uptake based on individual genetic patterns (33). Despite this, the clinical relevance of glycosylation remains limited in daily practice, perhaps due to the lack of comparative trials showing superior outcomes with tailored glycoform-based protocols.

In Section 2, focused on LH and FSH deficiency in assisted reproduction, a strong consensus emerged across multiple domains. In Domain 4, all three statements reached agreement, highlighting expert recognition of clinical indicators and risk factors for gonadotropin deficiency.

One of the key points supported by the panel was the use of E2 levels at the end of stimulation to determine the most appropriate method for triggering oocyte maturation. This approach received 73.3% agreement in the first round. Although the predictive value of basal E2 levels remains inconclusive (34), serum E2 at the end of stimulation is considered a useful indicator of granulosa cell activity. Historically associated with the development of ovarian hyperstimulation syndrome (OHSS), elevated E2 levels are now interpreted with caution. It is recognized that OHSS only occurs in the presence of high hCG levels, and that women with 17,20-desmolase deficiency may develop OHSS despite low E2 concentrations (35, 36). Therefore, increased E_2_ is now considered only as a marker of granulosa cell activity (37). Nonetheless, high or rapidly rising E2 levels remain valuable as markers of OHSS risk, particularly when levels exceed 2,500 pg/mL (38). Thus, assessing E2 at the end of stimulation can guide clinical decision-making to mitigate such risks. In addition to guiding triggering decisions, E2 levels at the end of stimulation have also been proposed as indicators for the optimal timing of oocyte retrieval (39), however findings differ among studies. Sarkar et al. (40) reported that high levels of E_2_ were linked with higher number of mature oocytes, zygotes exhibiting two pronuclei, cleavage stage embryos, blastocysts and vitrified embryos. Conversely, Palmerola et al. found no differences between low and normal E_2_ levels in oocyte fertilization and blastulation rates, and the percentage of normal embryos (41). Recently, an observational retrospective study reported that serum E_2_ levels could predict the number of retrieved metaphase II oocytes with limited accuracy (R^2^ = 0.232, p=0.000) (42), suggesting E2 may support oocyte maturity and fertilization but not pregnancy outcomes. These findings suggest that E2 levels at the end of stimulation may support various aspects of decision-making in assisted reproduction, particularly the choice and timing of triggering.

Another relevant aspect highlighted within Domain 4 was the interpretation of low E2 levels during stimulation with r-FSH as a sign of inadequate LH activity. This idea gained 76.2% agreement in the second round. Evidence supports that in GnRH agonist protocols with FSH monotherapy, endogenous LH is often suppressed, though residual levels may be sufficient in some cases to sustain follicular development and oocyte maturation (43). However, subgroups of patients may experience suboptimal responses due to insufficient LH stimulation. Clinical studies have shown that adding exogenous LH in these contexts can improve implantation and pregnancy outcomes, particularly in women previously treated with high doses of r-FSH (44). Since LH pulses stimulate E2 production in granulosa cells, persistently low E2 concentrations may reflect a deficiency in LH-mediated follicular activity (5). This interpretation is further supported by findings from Xu et al., who observed a correlation between E2 levels on the hCG trigger day and live birth rates (45), reinforcing the utility of E2 as a marker of follicular competence and adequate LH support. Overall, the available evidence supports the interpretation of low E2 levels as a reflection of insufficient LH activity during r-FSH stimulation, particularly in patients with suboptimal ovarian response.

Subsequently, 77.8% of participants in the first round agreed that long-term contraceptive methods might increase the risk of hypo-responsiveness in patients with associated risk factors. In the case of oral contraceptives, it has been proposed that either its progestogen compound might negatively affect endometrial receptivity or the provoked low LH levels might affect oocyte quality (46). In addition, the use of these contraceptive methods has been linked to a reduced ongoing pregnancy likelihood (46), and lower live birth rates (47). Barad et al. reported that hormonal contraceptives may impair follicle development by blocking gonadotropin support while allowing androgen exposure, causing initial growth followed by atresia due to low FSH levels (48). Finally, the use of contraceptive vaginal rings results have been associated with ovarian suppression (49).

In domain 6, participants highlighted advanced maternal age as a key factor aggravating gonadotropin deficiency. A significant majority (85.7% in the second round) agreed this deficiency is more pronounced in older women, consistent with findings by von Wolff et al., who reported significantly lower LH serum concentrations in women between 40–43 years compared to women between 28–34 years (50). Several studies have stated that an advanced maternal age might exacerbate the transient reduced LH and FSH production caused by GnRH analogues, leading to a sub-optimal response to ovarian stimulation (51–53). This decline in hormonal action is compounded by age-related decreases in the expression and sensitivity of FSH and LH receptors within ovarian cells, which limits the ovary’s capacity to respond effectively to gonadotropins (14). Concurrently, mitochondrial dysfunction plays a critical role in ovarian aging, as it reduces ATP production and increases mitochondrial damage in follicular cells and oocytes, impairing oocyte maturation and quality and decreasing follicular responsiveness to hormonal stimulation (54, 55).

This line of agreement continued into domain 7, where participants addressed hypo-response to ovarian stimulation due to reduced LH/FSH action. Notably, 71.4% of participants supported using the POSEIDON classification to individualize treatment in poor prognosis patients, enabling tailored strategies based on age, ovarian reserve markers, and prior stimulation response (56, 57). Additionally, r-LH supplementation was recommended for patients with unexpectedly low responses to r-FSH (73.8% agreement in the second round). This aligns with evidence showing that calibrated LH administration improves outcomes in patients over 35, with an initial abnormal ovarian response to r-hFSH, or with a low-prognosis after GnRH antagonist administration during the controlled ovarian stimulation (58). Supporting this, Verschuere et al. highlighted that LH supplementation might be beneficial in selected populations, such as women aged 36 to 39 or those with suboptimal responses to stimulation, although they also emphasized the current lack of robust evidence to define clear LH thresholds for clinical decision-making (22). Although a specific LH threshold to guide exogenous LH administration has not been established, several studies have nonetheless demonstrated improved outcomes in patients with suboptimal response to r-FSH, advanced maternal age, or increased risk of poor responsiveness under GnRH antagonist protocols (19). Recent meta-analyses confirm improved outcomes with r-LH supplementation in hypo-responders. Conforti et al. reported higher clinical pregnancy (OR: 2.03, p=0.003) and implantation rates (OR: 2.62, p=0.004), as well as a higher number of retrieved oocytes (weight mean differences: 1.98, p=0.03) compared to FSH monotherapy in this population (59).

These findings highlight strong clinical consensus that, despite limited guidelines, may guide the development of protocols for managing LH/FSH deficiency in assisted reproduction.

### 4.2 Statements without consensus achievement

Although nearly half of the statements reached consensus, 11 fell short of the 70% threshold, highlighting persistent uncertainty. These areas reflect domains with diverging expert opinions. While trends exist, variability prevents establishing standard practices.

A representative example is found in domain 1, concerning the transient severe LH deficiency induced by the administration of GnRH agonists/antagonists. Here, 48.9% and 66.7% of participants agreed across rounds that a transient hypogonadotropic state is triggered by the administration of these analogues. Although this did not meet the consensus criterion, the upward trend suggests growing awareness of the suppressive effects of GnRH analogues on LH secretion. It has been demonstrated that GnRH analogues can significantly reduce LH levels to prevent premature surges, a practice critical for cycle control (60, 61). However, whether this transient deficiency has clinical implications severe enough to justify therapeutic intervention remains contested.

Further divergence was evident in domain 3, addressing the clinical impact of genetic polymorphisms in LH/FSH and their receptors. While 66.7% of participants agreed that such polymorphisms might lead to poorer ovarian response, this figure did not meet the threshold for consensus. Interestingly, 40.5% of participants explicitly disagreed that these polymorphisms significantly increase the risk of low response in women with comorbidities. This divergence reflects the inconsistency found in literature. Among the most studied genetic variants are those in the FSH receptor gene, particularly rs6166 (Asn680Ser) and rs6165 (Thr307Ala) polymorphisms. The rs6166 polymorphism leads to the substitution of an asparagine by a serine, creating a potential phosphorylation site in the intracellular domain of the receptor. In rs6165, a threonine is substituted by alanine, leading to the loss of a potential glycosylation site (62, 63).

Carriers of the Ser680 allele typically require higher doses of FSH to reach E_2_ levels, produce fewer pre-ovulatory follicles, and have lower E_2_ levels on trigger day, indicating reduced receptor sensitivity (62, 64, 65). Moreover, a recent study by Baldini et al. reported that women with normal ovarian reserve markers (anti-Müllerian hormone (AMH) and antral follicle count (AFC)) but poor response to ovarian stimulation had a significantly higher prevalence of the Ser680 allele (66). These patients also showed reduced fertilization rates, indicating a potential functional defect in FSH receptor activity that is not reflected in baseline reserve markers. Similarly, Bosch et al. analyzed the impact of polymorphisms in the FSH and LH receptor genes in a general IVF population and found no overall difference in gonadotropin response. However, when stratified by AMH levels, carriers of the FSH receptor Ser680 variant with AMH below 15 pmol/L tended to yield fewer oocytes, suggesting that genetic effects may be more pronounced in women with low ovarian reserve (14). Regarding rs6165 polymorphism, it has also been associated with ovarian response, with AA homozygotes showing a shorter stimulation period and higher number of retrieved oocytes (63), as well as a greater number of obtained embryos (67). Conversely, a recent prospective study that analyzed the effect of several single nucleotide polymorphisms (SNPs) in the FSH receptor gene, including rs6166 and rs6165, found a reduction in the ovarian response to stimulation. However, the clinical relevance was limited, as patients remained as normal responders (68).

Meanwhile, beta variants of LH, studies have shown that it is common among hypo-responders, requiring an increased amount of r-FSH to achieve an optimal ovarian response during stimulation (69, 70). In addition, LH supplementation to women undergoing IVF based on their SNP profile (rs2293275) has been supported in a recent study, in which pregnancy and live birth rates were improved by administration of optimum levels of LH supplementation (71). However, the heterogeneity in genetic testing and its limited use in clinical workflows may explain why consensus was not reached among practitioners.

Within domain 5, which examined deficiency induced by the use of GnRH analogues, neither of the two statements included reached the 70% agreement threshold, though both showed a trend towards acceptance. The first statement considered the potential impact of GnRH analogue-induced LH/FSH suppression on folliculogenesis, steroidogenesis, and oocyte competence, received a 64.3% agreement in the second round. Animal studies support the concern that excessive suppression of endogenous gonadotropins may negatively affect clinical outcomes, demonstrating that GnRH analogues impact both folliculogenesis and steroidogenesis (72). This occurs because GnRH analogues suppress endogenous gonadotropin production by causing desensitization and downregulation of GnRH receptors in the pituitary gland, which decreases the synthesis and secretion of both LH and FSH (14). The reduction in circulating gonadotropins impairs the pituitary-ovarian axis, diminishing the signals necessary for normal follicular development and steroid hormone production. While residual LH after GnRH agonist administration is usually sufficient to support folliculogenesis when r-FSH is administered, some patients may still hyper-respond or experience deep LH suppression (73, 74), although folliculogenesis is not fully inhibited (75). In this regard, recent data by Verschuere et al., have shown that LH oversuppression following GnRH antagonist initiation is not only common, affecting over 45% of cycles, but also does not appear to be clearly detrimental to clinical outcomes, challenging the assumption that low LH levels are intrinsically harmful during stimulation (22).

The second statement proposed that GnRH analogues may increase hypo-response risk in patients with comorbidities, but consensus was again not reached. This may reflect difficulties isolating the effects of GnRH analogues from other variables. While the suppression of LH may contribute to suboptimal outcomes, the degree of impact likely differs based on the patient’s baseline characteristics and the stimulation protocol used. The degree of LH suppression varies across GnRH analogue protocols and can significantly influence ovarian response, particularly when using r-FSH (76). Antagonists induce a rapid and reversible suppression by competitively blocking GnRH receptors, while agonists cause an initial flare followed by profound downregulation. Although both are essential for preventing premature LH surges, they may paradoxically contribute to hypo-response, likely due to excessive LH suppression that fails to support optimal follicular growth despite exogenous FSH administration (14). This underscores the importance of individualizing stimulation protocols, including considering LH supplementation in selected patients (77, 78). The choice between antagonist and agonist protocols should be tailored to patient characteristics and clinical context, particularly in women with polycystic ovary syndrome (79). Furthermore, individual ovarian reserve and genetic polymorphisms in FSHR and FSHB genes may also modulate the ovarian response (68). These physiological and genetic differences likely contribute to the divergent opinions observed among Delphi participants regarding the impact of GnRH analogues.

In domain 6, addressing the effect of advanced maternal age on LH/FSH action, consensus was not reached on the combined use of r-hFSH:r-hLH to improve implantation, clinical pregnancy, and live birth rates in this population, despite 69% agreement, suggesting many clinicians see potential benefit. Evidence published in a recent meta-analysis by Conforti et al. reported that this combination significantly improved clinical pregnancy (OR: 1.45, p=0.03) and implantation rates (OR: 1.49, p=0.01) in women aged 35–40, though live birth rates remained unchanged (80). A large real-world study by Bielfeld et al. further supported these findings, reporting significantly higher clinical pregnancy (33.1% vs. 28.5%; *P*=0.001) and live birth rates (22.5% vs. 19.4%; *P*=0.014) with the combination of r-hFSH and r-hLH versus r-hFSH alone in women aged 35–40 with normal ovarian response (81). Additionally, recent findings suggest that combined FSH and LH therapy may potentially reduce mitochondrial fragmentation in aging cells by promoting mitochondrial fusion, enhancing mitochondrial length, mass, membrane potential, and ATP levels in mice (55).

The final group of statements without consensus belongs to domain 7, focused on hyporesponse to stimulation due to reduced LH/FSH action. This was one of the most contentious domains, with only two out of seven statements reaching consensus. Several indices have been proposed to assess ovarian sensitivity and classify poor responders, including the follicular output rate (FORT), follicle-to-oocyte index (FOI), and ovarian sensitivity index (OSI), which reflect the dynamic follicular response to exogenous gonadotrophins (82–84). These markers contrast with more static indicators of ovarian reserve like AFC and AMH. These indexes are known to be positively correlated with the outcomes of *in vitro* fertilization, as in the case of clinical pregnancy rates (83). However, Carosso et al. have recently reported that these markers are not useful in predicting live birth rates in advanced reproductive-aged women (85), who are usually more prone to a worse response to controlled ovarian stimulation (86, 87). In this Delphi project, most participants did not use FORT or FOI indexes to classify patients as low respondent (52.4% and 69.0% agreement in the second round, respectively). Nevertheless, participants stated their preference for the POSEIDON classification for predicting poor ovarian response (71.4% agreement in the second round). These criteria classify patients into four subgroups, considering the number of oocytes needed to obtain at least one euploid embryo for transfer, among other parameters (56). This approach allows for better identification and individualized management of patients with poor prognosis (57). Moreover, participants slightly supported the statement that the different gonadotropin schemes might affect endometrial receptivity (42.9% of agreement in the second round). GnRH analogue protocols have been reported to negatively affect endometrial receptivity during the controlled ovarian stimulation (88, 89). Early endometrial advancement with glandular maturation arrest is induced after stimulation with GnRH agonists and gonadotropins in the midluteal phase. Gonadotropin-mediated ovarian stimulation affects luteal phase function and alters endometrial receptivity. Additionally, there are notable differences between stimulated and natural cycles regarding periovulatory endometrial characteristics (89).

The panel did not reach consensus on whether r-hFSH:r-hLH is superior to hMG in patients with hypogonadotropic hypogonadism, and those with gonadotropin production/action deficiencies due to factors such as long-term contraception or advanced age. In both cases, the disagreement percentages exceeded 50%, indicating substantial resistance to favor recombinants over urinary gonadotropins. However, a randomized open-label study found that 88.0% of patients with hypogonadotropic hypogonadism were treated with a highly purified hMG and 70% of those treated with r-hFSH:r-hLH met the primary endpoint, having at least one follicle with a mean diameter of ≥17 mm, a pre-ovulatory serum E_2_ level of ≥400 pmol/l, and a mid-luteal phase serum P_4_ level of ≥25 nmol/l after 70 cycles, with no significant difference between treatments (p=0.11). Nevertheless, the combined treatment triggered more pregnancies than hMG across cycles, although differences between both groups were not statistically significant (58% vs 22%, p=0.06 in the first cycle; 57.1% vs 28.6%, p=0.42 in the second cycle; and 33.3% vs 18.2%, p=not available in the third cycle). When both the primary and secondary endpoints were analyzed, the r-hFSH:r-hLH treatment proved to be statistically superior to hMG (55.6% vs 23.3%, p<0.05) (90). Moreover, in populations other than those with hypogonadotropic hypogonadism, there is evidence supporting the superiority of r-hFSH:r-hLH over hMG. In women with extremely low ovarian reserve and poor response to stimulation (AFC < 4), r-hFSH:r-hLH was associated with significantly higher clinical pregnancy and implantation rates compared to hMG (91); other authors have reported that, among patients in whom more than 8 oocytes were retrieved, reflecting a subgroup with better ovarian response within a population of low-to-normal ovarian reserve, treatment with r-hFSH:r-hLH resulted in significantly higher pregnancy rates compared to hMG (92); and in an Asian population with normal ovarian reserve and normal response to stimulation, managed with a GnRH antagonist protocol, r-hFSH:r-hLH was associated with a higher cumulative live birth rate, lower miscarriage rate, and improved stimulation parameters compared to r-hFSH+hMG (93).

These disagreements reflect the complexity of reproductive endocrinology, where inconsistent evidence and clinical challenges limit consensus. They highlight how patient-specific factors often prevent universal treatment strategies.

Despite the results obtained, some factors and limitations should be taken into consideration when interpreting the present results. The cut-off level for agreement level at the present study, stated in 70%, is higher than other Delphi studies in the field (20) which established a 66% cut-off level. Although this approach ensured a high level of agreement, it may have limited the number of statements reaching consensus, especially those falling just below the threshold. However, a systematic review of consensus thresholds across various studies found a median value of 75%, which supports the selection of a 70% cut-off as a reasonable and methodologically sound choice (94). Additionally, the selection methodology of the participants and the email campaign, based on two attempts of spontaneous emails, may have affected response rates, with some experts missing the invitation or deadline. Furthermore, the agreement of these statements must be considered as the opinion of the participants, since experts consider individual patient characteristics when they choose the best treatment option for them. Nonetheless, despite these limitations, the use of a structured Delphi methodology and participation of experienced clinicians from varied institutions strengthen the validity of the results. The consensus statements obtained, though limited, offer valuable insights into areas of clinical agreement and can serve as a foundation for future guidance and decision-making in the management of LH and FSH deficiency in assisted reproduction.

## 5 Conclusions

The results obtained in this Delphi consensus highlight that a common strategy should be developed to establish an optimal management strategy for LH/FSH deficiency in women undergoing medically assisted reproduction. Participants mostly agreed on the relevance of LH/FSH deficiency due to a reduction of the action and production of gonadotropins and age in LH/FSH action, the relevance of E_2_ levels in the selection of the ovulation discharge technique, the use of POSEIDON to individualize the treatment of patient with poor prognosis, and the use of r-LH supplementation in patients with low responses. Overall, the diversity in the level of agreement of some statements, partially in line with the recent literature (14, 20), should be further investigated and might reflect the variability of the clinical practice in medically assisted reproduction. It is important to consider the diverse factors that can lead to an LH/FSH deficiency in order to optimize its management and improve reproductive outcomes.

## Data Availability

The raw data supporting the conclusions of this article will be made available by the authors, without undue reservation.
